# Effects of Lifestyle Measures, Antiobesity Agents, and Bariatric Surgery on Serological Markers of Inflammation in Obese Patients

**DOI:** 10.1155/2010/364957

**Published:** 2010-03-07

**Authors:** Konstantinos Tziomalos, Hariklia V. Dimitroula, Niki Katsiki, Christos Savopoulos, Apostolos I. Hatzitolios

**Affiliations:** First Propedeutic Department of Internal Medicine, Medical School, Aristotle University of Thessaloniki, AHEPA Hospital, 54646, Thessaloniki, Greece

## Abstract

Overweight and obesity are highly prevalent in developed countries and are also becoming more frequent in the developing world. Overweight and obese patients have elevated levels of several inflammatory markers and this inflammatory state might contribute to their increased vascular risk. We summarize the effects of lifestyle changes, antiobesity agents, and bariatric surgery on serological inflammatory markers in overweight and obese patients. Most studies showed a decrease in inflammation with all 3 interventions. However, it remains to be established whether the decrease in inflammatory markers induced by lifestyle changes or (where indicated) with antiobesity agents or bariatric surgery will translate into reduced vascular morbidity and mortality in overweight and obese patients.

## 1. Introduction

Overweight and obesity are highly prevalent in developed countries [1]. Approximately one third of the US population is obese and two thirds are overweight [1]. The prevalence of obesity is also increasing in developing countries [2]. This epidemic of overweight and obesity has major implications, since both overweight and obesity are independently associated with increased mortality [3–5]. 

Adipose tissue is not metabolically inert but synthesizes and secretes proinflammatory mediators, including interleukin 6 (IL-6) [6–9], tumor necrosis factor *α* (TNF*α*) [7, 10, 11], IL-8 [7], and monocyte chemoattractant [protein 1] (MCP-1) [12, 13]. Several studies reported that high sensitivity C-reactive protein levels are elevated in overweight and obese patients [14–26]. Compared with lean subjects, overweight and obese patients also have higher levels of IL-6 [9, 19, 24, 26–28], TNF*α* [24, 26–30], MCP-1 [27], as well as of other markers of inflammation, including IL-8 [31, 32], IL-18 [33], soluble TNF receptor-2 (sTNFR2) [27, 34, 35], soluble E-selectin [26, 36, 37], soluble intercellular adhesion molecule-1 (sICAM-1) [18, 26, 27, 34, 36–38], and soluble vascular cell adhesion molecule-1 (sVCAM-1) [26, 36]. In obese patients, inflammatory markers correlate with visceral adipose tissue mass [20, 21, 27, 33, 34, 39–41] and with waist circumference, an index of visceral adiposity [17, 21–23, 27, 32, 35, 39].

The activation of inflammation that is present in obesity might contribute to the increased vascular morbidity and mortality of these patients, since elevated levels of inflammatory markers are associated with increased vascular risk [42–46]. In this review, we summarize the effects of lifestyle changes, antiobesity agents, and bariatric surgery on serological inflammatory markers in obese patients.

## 2. Lifestyle Measures

Lifestyle measures are the cornerstone of the management of obesity [47]. In randomized controlled studies, diet and exercise reduced the risk of type 2 diabetes mellitus (T2DM) in overweight patients with impaired glucose tolerance (IGT) [48, 49]. In observational studies, weight loss achieved with lifestyle changes was associated with reduced incidence of coronary heart disease (CHD) [50] and with lower all-cause mortality [51]. 

Several studies evaluated the effects of diet and exercise alone or in combination on inflammatory markers in obese patients.

### 2.1. Diet Alone

Several small studies (*n* = 11–100) assessed the effects of low-calorie, very low-calorie, low-fat, or low-carbohydrate diets on inflammatory markers in obese patients (Table 1) [20, 22, 28, 52–75]. The duration of the intervention ranged from 3 weeks to 17 months [20, 22, 28, 52–75]. The majority of studies noted a significant decrease in CRP after weight loss induced by dietary modification in otherwise healthy overweight or obese adults [20, 22, 52, 54, 56–60, 62, 64–68, 70, 72–75], in obese patients with the metabolic syndrome [69], in overweight patients with raised triglycerides (TG) [55], in severely obese patients [71], or in obese patients with T2DM [53]. In some studies, the magnitude of decrease in hsCRP levels was linearly correlated to the amount of weight loss [20, 22, 67]. Only few reports did not record a significant fall in hsCRP concentration after weight loss [28, 63].

Furthermore, several studies compared the effects of different diets on inflammatory markers [52–54, 56, 57, 59, 62, 65–68, 70–73]. Heald et al. reported a significant 24% decrease in serum hsCRP levels after 3 months of replacement of high-fat foods with reduced-fat alternatives [59]. In another study, a carbohydrate restricted diet (CRD) with daily intake of eggs decreased hsCRP levels compared with a CRD without eggs, indicating that eggs significantly contribute to the anti-inflammatory effects of CRD possibly due to the antioxidant lutein which is present in eggs and modulates certain inflammatory responses [70]. A low-glycemic-load diet was also associated with a significantly greater improvement in serum hsCRP concentration compared with a low-fat diet (48 versus 5%) despite the similar weight loss in both diets [68]. In contrast, a more recent study reported a greater fall in hsCRP levels after a high-carbohydrate, low-saturated-fat diet compared with an isocaloric very-low-carbohydrate, high-saturated-fat diet, even though the latter group lost more weight and abdominal fat [62]. However, most studies comparing diets of different composition reported a similar decrease in hsCRP concentration among groups, suggesting that the weight loss *per se* rather than the dietary composition is the primary determinant of the fall in inflammatory markers during diet [52–54, 56, 57, 65–67, 71–73].

The effect of dietary intervention on other inflammatory markers is more controversial. Some studies reported a reduction in serum TNF*α* levels with diet [31, 72, 74, 76, 77] while others have not [28, 70, 75]. Similarly, serum IL-6 levels fell after diet-induced weight loss in some [28, 31, 72, 76], but not all studies [60, 74]. Regarding the effects of diet on IL-8, a significant reduction [76], increase [31], or no change [70] has been reported. In addition, caloric restriction did not affect circulating levels of the anti-inflammatory cytokine IL-10 [78]. However, limited data suggest that diet-induced weight loss is associated with a proportional reduction in IL-18 [33] and IL-20 levels [79]. Weight loss by caloric restriction also induces significant decreases in sICAM-1 [36, 52, 55, 61, 62, 72] and sVCAM-1 levels [36, 61, 80]. Only one study reported no change in sICAM-1 and sVCAM-1 levels [70] and another study reported an increase in sVCAM-1 levels [62].

The conflicting results mentioned above could be attributed to the differences in baseline characteristics of the studied population that might influence inflammatory markers (i.e., age, gender and coexisting diseases), the brief duration of the intervention, the insufficient sample size, or the degree of body weight reduction. Overall, the greatest reductions in serological markers of inflammation were observed in studies where an at least 10% weight loss was achieved.

Interestingly, recent studies demonstrated that brief periods of reduced food intake can increase resistance to ischemia reperfusion injury in rodents and modify circulating levels of pro-inflammatory agents in humans [81, 82]. The effects of short-term interventions on inflammation appear to be mostly due to the calorie restriction and negative energy balance whereas in long-term interventions weight loss and adipose mass reduction also contribute to the suppression of inflammation. However, existing studies do not show a major difference between the effects of short and long terms of calorie restriction on the levels of inflammatory markers. The 2 studies that did not report an effect of diet on hsCRP levels lasted for 3 weeks and 3 months, respectively [28, 63]. In addition, both short- and long-lasting dietary interventions (3 weeks to 17 months) induced a reduction in hsCRP levels in several studies [20, 22, 52–60, 62, 64–75].

### 2.2. Exercise Alone

A number of small studies (*n* = 8–199) evaluated the effects of exercise training, resistance training, or aerobic exercise training for 2–12 months on inflammatory markers (Table 1) [35, 83–93]. Some [83–87] but not all [88–90] studies reported a significant reduction in serum hsCRP levels. Interestingly, body weight changed only slightly in some studies that reported a significant fall in hsCRP levels [83–85]. Limited data exist on the impact of exercise on other markers of inflammation. Serum T*Ν*F*α* levels fell after aerobic exercise training in most [35, 87, 91] but not all studies [84]. In contrast, serum IL-6 levels did not change with exercise in most reports [83, 85, 89] and fell only in 1 study [90]. The concentration of IL-10, an anti-inflammatory cytokine, increased after 6 months of aerobic exercise in overweight patients with T2DM [84] and serum IL-18 concentration decreased in the same study [84], but remained unchanged in another [93]. In addition, sICAM-1 and sVCAM-1 levels were not affected after one year of resistance training [85]. In a recent controlled exercise training intervention, 189 overweight and mildly obese patients were randomized to 6 months of inactivity or 1 of 3 types of aerobic exercise training regimens: low-amount/moderate intensity, low-amount/vigorous intensity, and high-amount/vigorous intensity [92]. Despite a mean reduction of adipose tissue mass by 9 ± 11% (or 2.5 ± 3.0 kg), exercise training did not affect hsCRP, IL-6, or T*Ν*F*α* levels to a significant degree [92].

Differences in the type, frequency, intensity, and duration of exercise, in the amount of achieved weight loss, and/or in the duration of the study may partly explain the observed discrepancies in the effects of exercise on markers of inflammation. Moreover, the participants were mainly overweight or mildly obese, with variable baseline characteristics (age, gender, previous physical activity, all of which might have influenced the results). Finally, it has been reported that both strenuous exercise (which can lead to muscle damage) and overtraining (which might induce oxidative stress) might act as an inflammatory stimulus and raise inflammatory markers [86].

### 2.3. Combination of Diet and Exercise

More data are available on the effects of combining both diet and exercise on inflammatory markers (Table 1) [26, 29, 40, 94–114]. All studies (*n* = 12–190, duration 4 weeks to 2 years) reported a significant decrease in BMI in overweight [94, 111, 112], obese [26, 29, 40, 95–107, 109, 111, 112] and morbidly obese patients [110, 113, 114]. Interestingly, hsCRP levels significantly decreased in all but 1 of these studies [26, 29, 40, 94–96, 98–114]; the remaining study reported a nonsignificant fall in hsCRP concentration (*P* = .71) [97]. In accordance with diet-only studies, hsCRP reductions after diet and exercise correlated positively with changes in body weight and fat mass [101]. The majority of studies also showed a significant decrease in serum IL-6 concentration after a multidisciplinary program including diet and exercise [26, 94, 97, 98, 100–103, 107, 110–113] and only few studies did not [95, 96, 109]. Furthermore, all studies that evaluated the effects of caloric restriction and exercise on serum IL-18 levels observed a significant decrease [100–102, 106, 111]. Limited data also suggest that diet and increased physical activity decrease IL-7 [111], IL-8 [113], sICAM-1 [26, 104], and sVCAM-1 levels [26]. In contrast, the effects of diet and exercise on serum TNF*α* levels were less consistent with both significant decreases [26, 40, 101, 105] and no change reported [94, 95, 97, 109, 112].

Finally, 4 studies assessed the separate and combined effects of exercise and diet intervention on several cytokines. Nicklas et al. reported that an 18-month dietary intervention resulted in significant reductions in hsCRP and IL-6 concentrations (but not in TNF*α* levels) whereas exercise training had no effect; however, weight loss was observed only in patients on diet [115]. Patients on both diet and exercise did not show greater reductions in hsCRP and IL-6 levels than those on diet alone [115]. In another study, 6 months of diet plus exercise, but not exercise alone, decreased hsCRP and IL-6 levels (but not TNF*α* levels) in obese postmenopausal women despite a similar weight and fat loss in both groups [116]. Rokling-Andersen et al. randomly allocated 188 men with vascular risk factors to 4 groups: diet, exercise, combined diet and exercise, and control [117]. Serum TNF*α* levels increased significantly in the 3 intervention groups but the increase was smaller in the combined diet and exercise group and probably represents a chance finding [117]. In another study, 33 obese postmenopausal women with T2DM were assigned to diet, exercise, or diet and exercise for 14 weeks. Serum hsCRP levels decreased by approximately 15% with all interventions even though weight loss was observed only in patients assigned to diet alone or with exercise [118]. In contrast, serum TNF*α* levels did not change significantly [118].

The conflicting effects of the combination of diet and exercise on inflammatory markers are probably due to the short duration of the majority of the studies, the small number of participants, or the varying weight loss achieved. The lack of a decrease in serum TNF*α* concentration may be a result of its transient production and short half-life. Similar to the studies that assessed dietary modifications alone, a weight loss of at least 10% resulted in the greatest improvements in serological markers of inflammation in studies where diet was combined with exercise.

Overall, diet and exercise alone or in combination have favourable effects on serum inflammatory markers. The inconsistent findings in some studies may be due to (apart from the reasons already mentioned) differences in the studied population (i.e., overweight, obese, patients with diabetes), in the baseline concentration of cytokines and inflammatory status of each patient, as well as in the laboratory methods used for the measurement of cytokines.

## 3. Antiobesity Agents

Orlistat induces weight loss by reducing intestinal fat absorption (by up to 30%) by binding to pancreatic lipases and partially inhibiting the hydrolysis of dietary fat (triglycerides) into absorbable free fatty acids and monoacylglycerols [119]. Compared with placebo, orlistat reduces body weight by approximately 2.9 kg (95% confidence interval (CI) 2.5–3.2 kg) in studies lasting more than 1 year [120]. In addition, orlistat lowers low density lipoprotein cholesterol (LDL-C) levels, reduces blood pressure (BP), and improves glycemic control in diabetic patients [120, 121]. More importantly, in XENical in the prevention of Diabetes in Obese Subjects (XENDOS) study, orlistat reduced the risk of developing T2DM in obese patients with impaired glucose tolerance [122].

A limited number of studies evaluated the effects of orlistat on inflammatory markers in obese patients (Table 1). In some reports, treatment with orlistat for up to 1 year reduced hsCRP levels [123–125]. However, in other studies the fall in CRP levels during orlistat treatment did not reach statistical significance [126, 127]. In addition, in a placebo-controlled study that evaluated the effects of long-term (3 years) treatment with orlistat, hsCRP levels decreased significantly only in patients who lost more than 11% of their body weight [128]. It is of interest to point out that all 3 studies that reported a decrease in hsCRP levels during orlistat treatment [123–125] lasted for 6 months or more and hsCRP levels were measured. In contrast, negative studies evaluated CRP levels, which are less sensitive for evaluating inflammation than hsCRP [126, 127]. Moreover, baseline CRP levels were within the normal range in both negative studies and this might have limited their statistical power to show a significant decrease in CRP levels [126, 127]. Finally, both negative studies had shorter follow-up than the studies that reported a decrease in hsCRP levels during orlistat treatment (3 and 4 months, resp.) and the potential antiinflammatory effect of orlistat might have not become apparent yet at study completion [123–127].

A reduction in serum IL-6 [123, 124, 129] and TNF*α* levels was also reported after treatment with orlistat [123, 127, 129]. Orlistat did not affect serum IL-6 levels only in one study, which however had a short follow-up as already mentioned (4 months versus 6 months or more in other reports) [127]. Interestingly, the decrease in hsCRP and TNF*α* in some studies was greater in the diet plus orlistat group compared with patients who were only on diet even after correcting for the greater weight loss observed in the orlistat group [123, 125, 129]. Overall, it appears that 6 months or more of treatment with orlistat and a weight loss greater than 10% are required for a significant reduction in inflammatory markers with this agent. 

Sibutramine lowers body weight by increasing satiety and by reducing appetite through the selective inhibition of the neuronal reuptake of serotonin and noradrenaline within the hypothalamus [130, 131]. In studies lasting more than 1 year, sibutramine reduced body weight by approximately 4.2 kg (95% CI 3.6–4.7 kg) compared with placebo [120, 130]. Sibutramine also lowers triglyceride (TG) levels but increases BP and pulse rate [120, 130].

In some studies, sibutramine reduced serum levels of hsCRP [132, 133], IL-6, and TNF*α* and increased serum levels of IL-10 when given for 3–6 months (Table 1) [134]. A weight loss of 6.1–7.1 kg (5.4%–6.9%) was observed during the latter studies [133, 134] and correlated with the reduction in IL-6 levels [134]. In another study, treatment with sibutramine for 6 months did not affect hsCRP levels; however, the weight loss achieved in this study was substantially smaller (2.4 kg or 2.9%) [135]. Therefore, similarly with orlistat, weight loss appears to be the major factor that determines the anti-inflammatory effect of sibutramine.

In a nonrandomized, nonblinded comparative study, only sibutramine reduced hsCRP levels after 6 months whereas changes in hsCRP levels were nonsignificant in patients given orlistat [133]. However, weight loss was greater in the sibutramine group compared with the orlistat group (5.4% versus 2.5%, resp.), suggesting again that the degree of weight loss correlates with the anti-inflammatory action of these antiobesity agents [133]. Whether orlistat or sibutramine exerts an anti-inflammatory action independently of weight loss is unclear.

It should emphasized that the Committee for Medicinal Products for Human Use of the European Medicines Agency recently concluded that the risks of sibutramine are greater than its benefits and recommended the suspension of marketing authorization of this agent across the European Union (http://www.ema.europa.eu/pdfs/human/referral/sibutramine/3940810en.pdf; accessed 25/1/2010). The Food and Drug Administration also stated that sibutramine is not to be used in patients with a history of coronary heart disease, stroke or transient ischemic attack, heart failure, heart arrhythmias, peripheral arterial disease, or uncontrolled hypertension (http://www.fda.gov/Safety/MedWatch/SafetyInformation/SafetyAlertsforHumanMedicalProducts/ucm198221.htm; accessed 25/1/2010). These recommendations were based on the results of the Sibutramine Cardiovascular Outcome Trial (SCOUT), which showed an increased risk of nonfatal cardiovascular events with sibutramine compared with placebo in patients with known or high risk for cardiovascular disease (http://www.ema.europa.eu/pdfs/human/referral/sibutramine/3940810en.pdf and http://www.fda.gov/Safety/MedWatch/SafetyInformation/SafetyAlertsforHumanMedicalProducts/ucm198221.htm; accessed 25/1/2010).

Metformin enhances insulin sensitivity, lowers hepatic glucose production, and is a first-line treatment for T2DM [136, 137]. Metformin frequently induces a modest weight loss in diabetic patients [136]. In 4 studies in patients with T2DM, treatment with metformin for 3-4 months exerted conflicting effects on serum hsCRP, IL-6, TNF*α*, sICAM-1, sVCAM-1, and soluble E-selectin levels (Table 1) [138–141]. In one study where metformin was administered only for 1 month, no significant change was observed in hsCRP levels [142]. Metformin is also used in the management of patients with polycystic ovary syndrome (PCOS), where it has been shown to ameliorate reproductive abnormalities, restore ovulation and regular menses, increase pregnancy rates, and reduce androgen production [143]. In 3 studies in women with PCOS, treatment with metformin for 6 months also had contradictory effects on hsCRP, IL-6, TNF-*α*, sICAM-1, sVCAM-1, and soluble E-selectin levels (Table 1) [144–146]. In addition to the unclear effects of metformin on inflammatory markers, it should be emphasized that metformin is not currently recommended for the management of obesity. Moreover, a recent study in nondiabetic obese women showed that sibutramine monotherapy reduced hsCRP levels and that adding metformin to sibutramine did not result in any additional decrease in hsCRP levels [147].

## 4. Bariatric Surgery

Bariatric surgery is classified in restrictive (laparoscopic adjustable gastric banding and vertical banded gastroplasty), malabsorptive (biliopancreatic diversion with or without duodenal switch), and combination procedures (Roux-en-Y gastric bypass) [148]. All forms of bariatric surgery result in loss of more than 50% of excess weight [149]. In addition, hypertension, T2DM, hyperlipidemia, and obstructive sleep apnea resolve in the majority of patients who undergo bariatric surgery [149]. More importantly, bariatric surgery was associated with reduced all-cause mortality in several retrospective studies [150–152] and in a prospective study [153]. Malabsorptive or combination procedures induce greater weight loss [152, 154–157] and appear to be more effective in the resolution of obesity-associated comorbidities [149, 157]. However, they are also associated with higher complication rates compared with restrictive procedures [149, 151, 154].

Several studies reported a reduction in serum hsCRP levels after Roux-en-Y gastric bypass (Table 1) [158–166]. This decrease in hsCRP levels is greater in patients who lost more weight after surgery [164, 166]. A decline in hsCRP levels also occurs in patients undergoing gastric banding (Table 1) [158, 167–175]. The decrease in hsCRP levels correlated with the fall in BMI in some of these studies [172]. Only one study did not report a significant change of hsCRP levels after gastric banding; however, the decrease in hsCRP levels in this report was significant 6 months after surgery (*P* = .05) and there was a trend for significant decrease also at 12 months (*P* = .08) [176]. In a recent study, patients who underwent gastric bypass lost more weight than those who underwent gastric banding but the fall in hsCRP levels was similar in both groups [158]. Other less frequently performed bariatric procedures, such as biliopancreatic diversion [177], laparoscopic sleeve gastrectomy [178], and gastric partition [179], also result in a decrease in hsCRP levels that in some cases correlates with the reduction in BMI [178]. The reduction in hsCRP levels in almost all studies of all types of bariatric surgery, in contradiction to the equivocal results of lifestyle changes and antiobesity agents, further supports the notion that weight loss *per se* is the main factor that causes the fall in hsCRP levels, since bariatric surgery results in substantially greater weight loss than lifestyle changes or antiobesity agents. The lack of correlation between weight loss and hsCRP fall might be attributed to the small number of patients included in these studies, which limits their statistical power.

Other markers of inflammation, including IL-6 [160, 162, 163, 165], IL-18 [180], MCP-1 [180], and soluble E-selectin [166] also fall after gastric bypass (Table 1). Only 1 small study (*n* = 26) did not report significant changes in IL-6, TNF*α*, sTNFR-2, sICAM-1, and sVCAM-1 levels following gastric bypass [166]. However, serum hsCRP levels, which are a more sensitive and specific marker of inflammation, decreased in this study [166]. Serum levels of IL-6 [171, 172], IL-18 [167], sTNFR-2 [167, 170], MCP-1 [167], soluble E-selectin [37, 176, 181, 182], sICAM-1 [37, 181, 182], and sVCAM-1 levels [176] also fall after gastric banding (Table 1). The change in soluble E-selectin and sICAM-1 levels correlated with the reduction in BMI [37, 182]. However, IL-6 [174], sVCAM-1 [181], and sICAM-1 levels [176] did not change significantly in other studies. In addition, serum TNF*α* levels [172, 174] were not affected by gastric banding. Again, serum hsCRP levels fell in most studies that did not record a significant change in other inflammatory markers [172, 174], suggesting that the latter might be less sensitive in identifying a clinically significant anti-inflammatory effect of bariatric surgery.

In contrast to bariatric surgery, removal of abdominal subcutaneous fat by liposuction does not appear to reduce hsCRP, IL-6, or TNF*α* levels (Table 1) [183, 184]. However, one study reported a reduction in serum hsCRP, IL-6, IL-18, and TNF*α* levels after liposuction [185]. In the latter study, the amount of fat aspirate correlated with the reduction in TNF*α* levels [185]. Even though the data on the effect of liposuction on inflammatory markers are limited, the predominantly negative findings are concordant with the concept that visceral adipose tissue is the main factor inducing the inflammatory milieu of obese patients whereas subcutaneous adipose tissue is less important.

## 5. Conclusions

Existing evidence suggests that lifestyle measures, antiobesity agents, and bariatric surgery reduce serum levels of inflammatory markers in obese patients. The lack of significant change in some studies appears to be due to small sample size and/or small weight loss achieved. It appears that the reduction in inflammation is primarily driven by weight loss [186]. In a recent meta-analysis, a linear relation was observed between weight loss following lifestyle changes or bariatric surgery and the fall in hsCRP levels, which declined by 0.13 mg/L for each 1 kg of weight loss [186]. What remains to be established is whether reducing inflammatory markers in obese patients with lifestyle measures or (where indicated) with bariatric surgery or drugs will translate in lower vascular risk. In this context, a beneficial effect of suppressing inflammation has been reported during statin treatment in patients with or without established vascular disease, even though it was not assessed whether obese patients benefit more or less [187–189].

## Figures and Tables

**Table 1:** Effects of lifestyle measures, antiobesity agents, and bariatric surgery on serological markers of inflammation in obese patients.

Intervention	Marker of inflammation	Effect	Reference
Diet alone	hsCRP	↓	↓: [20, 22, 52–60, 62, 64–75, 115, 118]
		or no effect	
			no effect: [28, 63]
	TNF*α*	↓	↓: [31, 72, 74, 76, 77]
		or ↑	↑: [117]
		or no effect	no effect: [28, 70, 75, 115, 118]
	IL-6	↓	↓: [28, 31, 72, 76, 115]
		or no effect	no effect: [60, 74]
	IL-8	↓	↓: [76]
		or ↑	↑: [31]
		or no effect	no effect: [70]
	IL-10	No effect	[78]
	IL-18	↓	[33]
	IL-20	↓	[79]
	sICAM-1	↓	↓: [36, 52, 55, 61, 62, 72]
		or no effect	no effect: [70]
	sVCAM-1	↓	↓: [36, 61, 80]
		or ↑	↑: [62]
		or no effect	no effect: [70]

Exercise alone	hsCRP	↓	↓: [83–87]
		or no effect	no effect: [88–90, 92, 115, 116, 118]

	TNF*α*	↓	↓: [35, 87, 91]
		or ↑	↑: [117]
		or no effect	no effect: [84, 92, 115, 116, 118]
	IL-6	↓	↓: [90]
		or no effect	no effect: [83, 85, 89, 92, 115, 116]

	IL-10	↑	[84]
	IL-18	↓	↓: [84]
		or no effect	no effect: [93]
	sICAM-1	No effect	[85]
	sVCAM-1	No effect	[85]

Combination of diet and exercise	hsCRP	↓	↓: [26, 29, 40, 94–96, 98–116, 118]

		or no effect	no effect: [97]
	TNF*α*	↓	↓: [26, 40, 101, 105]
		or ↑	↑: [117]
		or no effect	no effect: [94, 95, 97, 109, 112, 115, 116, 118]

	IL-6	↓	↓: [26, 94, 97, 98, 100–103, 107, 110–113, 115, 116]
		or no effect	
			no effect: [95, 96, 109]
	IL-7	↓	[111]
	IL-8	↓	[113]
	IL-18	↓	[100–102, 106, 111]
	sICAM-1	↓	[26, 104]
	sVCAM-1	↓	[26]
Orlistat	hsCRP	↓	↓: [123–125, 128]
		or no effect	no effect: [126, 127, 133]
	TNF*α*	↓	[123, 127, 129]
	IL-6	↓	↓: [123, 124, 129]
		or no effect	no effect: [127]

Sibutramine	hsCRP	↓	↓: [132, 133, 147]
		or no effect	no effect: [135]
	TNF*α*	↓	[134]
	IL-6	↓	[134]
	IL-10	↑	[134]

Metformin	hsCRP	↓	↓: [139, 141, 144, 146, 147]
		or no effect	no effect: [140, 142, 145]
	TNF*α*	No effect	[139, 145]
	IL-6	↓	↓: [144]
		or no effect	no effect: [139, 145]
	sICAM-1	↓	↓: [139]
		or no effect	no effect: [138, 140, 146]
	sVCAM-1	↓	[138–140, 146]
	soluble E-selectin	↓	↓: [139, 140]
		or no effect	no effect: [138, 146]

Roux-en-Y gastric bypass	hsCRP	↓	[158–166]
	TNF*α*	No effect	[166]
	sTNFR-2	No effect	[166]
	IL-6	↓	↓: [160, 162, 163, 165]
		or no effect	no effect: [166]
	IL-18	↓	[180]
	sICAM-1	No effect	[166]
	sVCAM-1	No effect	[166]
	soluble E-selectin	↓	[166]
	MCP-1	↓	[180]

Gastric banding	hsCRP	↓	↓: [158, 169–175]
		or no effect	no effect: [176]
	TNF*α*	No effect	[172, 174]
	sTNFR-2	↓	[167, 180]
	IL-6	↓	↓: [171, 172]
		or no effect	no effect: [174]
	IL-18	↓	[167]
	sICAM-1	↓	↓: [37, 171, 182]
		or no effect	no effect: [176]
	sVCAM-1	↓	↓: [176]
		or no effect	no effect: [181]
	soluble E-selectin	↓	[37, 176, 181, 182]
	MCP-1	↓	[167]

Liposuction	hsCRP	No effect	No effect: [183, 184]
		or ↓	↓: [185]
	TNF*α*	No effect	No effect: [183, 184]
		or ↓	↓: [185]
	IL-6	No effect	No effect: [183, 184]
		or ↓	↓: [185]
	IL-18	↓	[185]

hsCRP: high sensitivity C-reactive protein; IL: interleukin; TNF*α*: tumor necrosis factor *α*; sICAM-1: soluble intercellular adhesion molecule 1; sVCAM-1: soluble vascular cell adhesion molecule 1; sTNFR-2: soluble TNF receptor 2.
